# Monoclonal Gammopathy of Renal Significance: Clinical and Histological Efficacy of a Bortezomib-Based Regimen

**DOI:** 10.3389/fmed.2020.587345

**Published:** 2020-12-16

**Authors:** Giacomo Quattrocchio, Antonella Barreca, Antonella Vaccarino, Giulio Del Vecchio, Emanuele De Simone, Roberta Fenoglio, Michela Ferro, Maria Pagliaro, Massimo Pini, Massimo Manes, Dario Roccatello

**Affiliations:** ^1^Nephrology and Dialysis Unit, San Giovanni Bosco Hospital, Turin, Italy; ^2^Division of Pathology, Città della Salute e della Scienza Hospital, Turin, Italy; ^3^Hematology and Thrombotic Diseases, San Giovanni Bosco Hospital, Turin, Italy; ^4^Nephrology and Dialysis Unit, Umberto Parini Hospital, Aosta, Italy; ^5^Center of Research of Immunopathology and Rare Diseases (CMID), Department of Rare, Immunologic, Hematologic and Immunohematologic Diseases, San Giovanni Bosco Hospital, University of Turin, Turin, Italy

**Keywords:** monoclonal gammopathy of renal significance (MGRS), C3 glomerulonephritis (C3GN), Immunotactoid glomerulonephritis, bortezomib, glomerulonephritis

## Abstract

Monoclonal Gammopathy of Renal Significance (MGRS) is a group of heterogeneous disorders characterized by renal dysfunction secondary to the production of a monoclonal immunoglobulin by a nonmalignant B cell or plasma cell clone. We report the clinical and histological outcomes of two patients with biopsy-proven MGRS: one patient showed membranoproliferative glomerulonephritis with monoclonal k-light chain and C3 deposits, the second patient showed immunotactoid glomerulopathy. Both patients were treated with a 9-month chemotherapy protocol including bortezomib, cyclophosphamide, and dexamethasone. Renal biospy was repeated after 1 year. The estimated glomerular filtration rate (eGFR) increased from 22.5 (baseline) to 40 ml/min per 1.73 m2 after 12 months, then to 51.5 ml/min per 1.73 m2 after 24 months; proteinuria decreased from 4.85 (baseline) to 0.17 g/day after 12 months, then to 0.14 g/day after 24 months. Repeat renal biopsies showed a dramatic improvement of the glomerular proliferative lesions and near complete disappearance of the immune deposits. A bortezomib-based treatment proved very effective and was well-tolerated in the two patients presenting with clinically and histologically aggressive MGRS.

## Introduction

The term monoclonal gammopathy of renal significance (MGRS) was proposed for the first time in 2012 by the International Kidney and Monoclonal Gammopathy Research Group (IKMG) to describe patients with renal injury secondary to a plasma cell clone who do not present the criteria for multiple myeloma or a lymphoproliferative disorder (1). The hematologic disorder is generally consistent with monoclonal gammopathy of undetermined significance (MGUS) (2).

In 2017 the IKMG updated the term MGRS and redefined it as a clonal proliferative disorder that produces a nephrotoxic monoclonal immunoglobulin and does not meet previously defined haematologic criteria for the treatment of a specific malignancy. Furthermore, the IKMG recommended kidney biopsy for the correct diagnosis of MGRS-related disease (3). Renal disease can result from the direct deposition of nephrotoxic monoclonal immunoglobulin (MIg) or its light- or heavy-chain fragments in various renal tissue compartments, and includes common disorders, such as cast nephropathy, amyloidosis, and MIg deposition diseases, as well as rarer disorders, such as immunotactoid glomerulopathy, proliferative GN with MIg deposits, light-chain proximal tubulopathy, and the rare entities of crystal-storing histiocytosis and crystalglobulinemia. MGRS can also result from indirect mechanisms and manifests as C3 glomerulopathy or thrombotic microangiopathy without tissue MIg deposits (4).

Effective chemotherapy regimens as well as stem cell transplantation are widely used for renal complications of myeloma kidney and amyloid light-chain amyloidosis (5, 6). Specific therapeutic protocols for MGRS are far less well-defined and some reluctance exists among hematologists to treat this disorder. However, both the progressive nature of MGRS and its tendency to recur after kidney transplantation strongly suggest—at least in patients with severe disease—treatment which should target the pathologic clone responsible for the production of the nephrotoxic MIg (7).

Herein we report the clinical outcome and the histological renal evolution of two patients with two different monoclonal Ig-associated diseases at renal biopsy who were treated with a combination regimen including bortezomib, dexamethasone, and cyclophosphamide.

## Case Presentation

### Case 1

A 59 year old white female with a history of essential hypertension treated with ramipril was referred to us for a second opinion by another hospital where she had undergone renal biopsy for renal failure (serum creatinine level 1.5 mg/dl), proteinuria (2.9 g/d), and microscopic dysmorphic hematuria. The renal biopsy had shown membranoproliferative glomerulonephritis with C3 and k-light chain deposits involving glomerular capillary walls and the mesangium. Unfortunately, the histological specimen was not suitable for adequate electron microscopy study and full immunofluorescence analysis.

At admission to our hospital she referred asthenia and showed mild lower limb edema. Laboratory investigations showed (Table 1): reduced estimated glomerular filtration rate (eGFR) (35 ml/min per 1.73 m2), severe proteinuria (2.7 g/d) and mild microscopic hematuria; normal calcemia, immunoglobulin and complement levels; negative cryoglobulins; normocytic anemia (hemoglobin, 9.4 g/dl); negative serum immunofixation with moderately increased serum k/λ free light chain concentration (k/λ ratio = 11.5), and scanty amounts of k free light chain in urine immunofixation.

**Table 1:** Clinical, histological, and laboratory features of patients.

	**Patient 1**	**Patient 2**
Age (yr)	59	71
Sex	Female	Male
Kidney histology	PGNMID	ITGN
Bone marrow plasma cells (%)	7	6
Baseline eGFR (ml/min per 1.73m^2^)	35	10
12-month eGFR (ml/min per 1.73m^2^)	49	31
24-month eGFR (ml/min per 1.73m^2^)	57	46
Baseline proteinuria (g/day)	2.7	7.0
12-month proteinuria (g/day)	0.09	0.26
24-month proteinuria (g/day)	0.05	0.23
Baseline Hemoglobin (g/dL)	9.4	10.5
12-month Hemoglobin (g/dL)	12.8	13.1
24-month Hemoglobin (g/dL)	13.5	17.0
Baseline serum Calcium (mmol/L)	2.2	1.9
12-month serum Calcium (mmol/L)	2.5	2.3
24-month serum Calcium (mmol/L)	2.5	2.3
Baseline k/λ sFLC (mg/l)	369/32	89/41
12-month k/λ sFLC (mg/l)	23/16	16/14
24-month k/λ sFLC (mg/l)	31/17	19/16

*yr, year; PGNMID, proliferative glomerulonephritis with monoclonal Ig deposits; ITGN, immunotactoid glomerulonephritis; eGFR, estimated glomerular filtration rate; k/λ sFLC, k/λ serum free light chain concentration*.

A bone marrow aspirate and biopsy were performed and a real-time ultrasound-guided percutaneous renal biopsy was repeated.

Bone marrow aspirate and biopsy were examined by light microscopy, immunohistochemistry, and flow cytometry, and only showed 7% polyclonal plasma cells.

**Figure 1:**
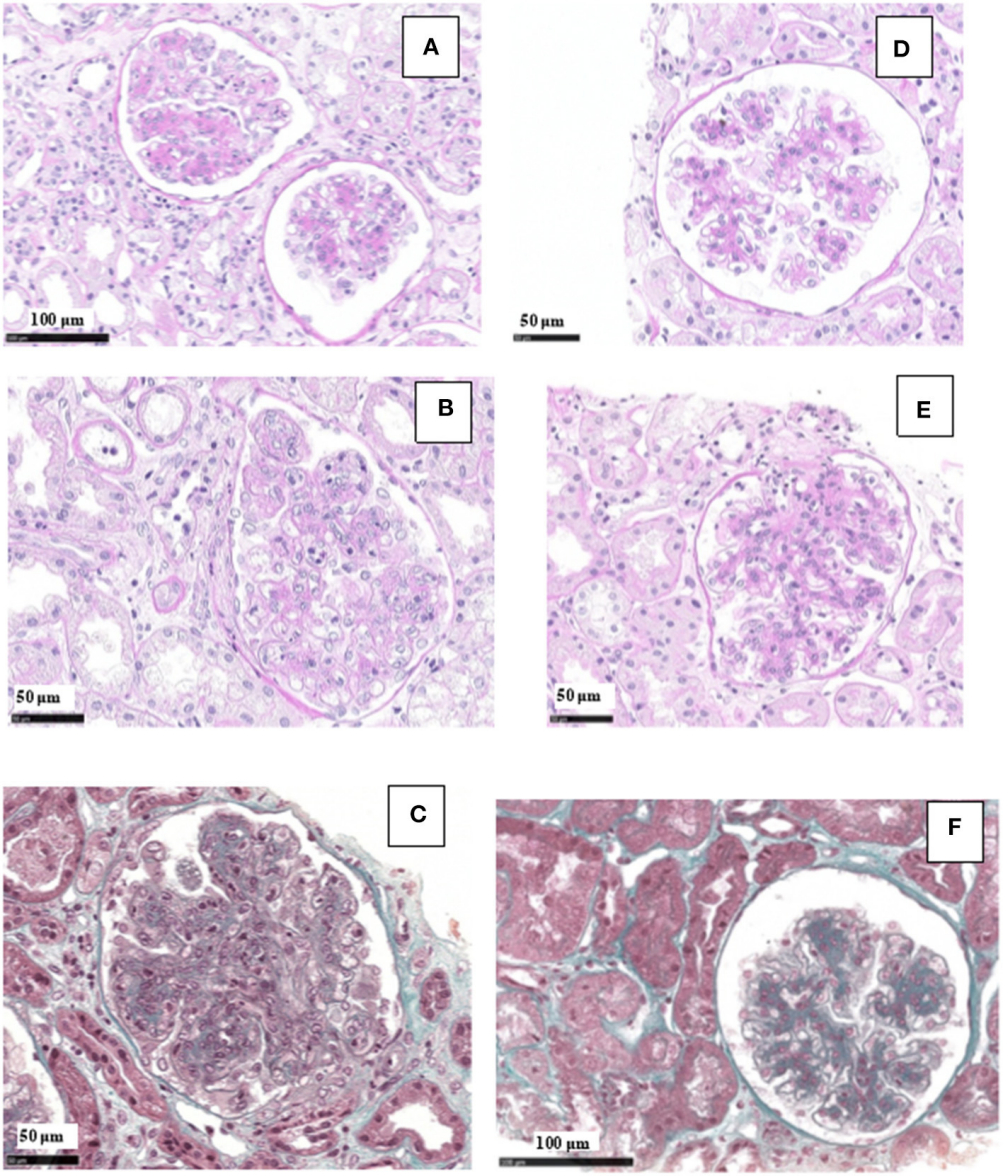
Light microscopy findings in patient #1: first biopsy showed membranoproliferative glomerulonephritis with focal mesangial nodules, and endocapillary mononuclear and polymorphonuclear leukocytes (**A**, PAS; **B**, PAS; **C**, Masson Trichrome). The repeat biopsy showed a remarkable reduction of the membranoproliferative lesions with disappearance of the capillary leukocyte infiltration and persistent mesangial expansion (**D**, PAS; **E**, PAS; **F**, Masson Trichrome).

**Figure 2:**
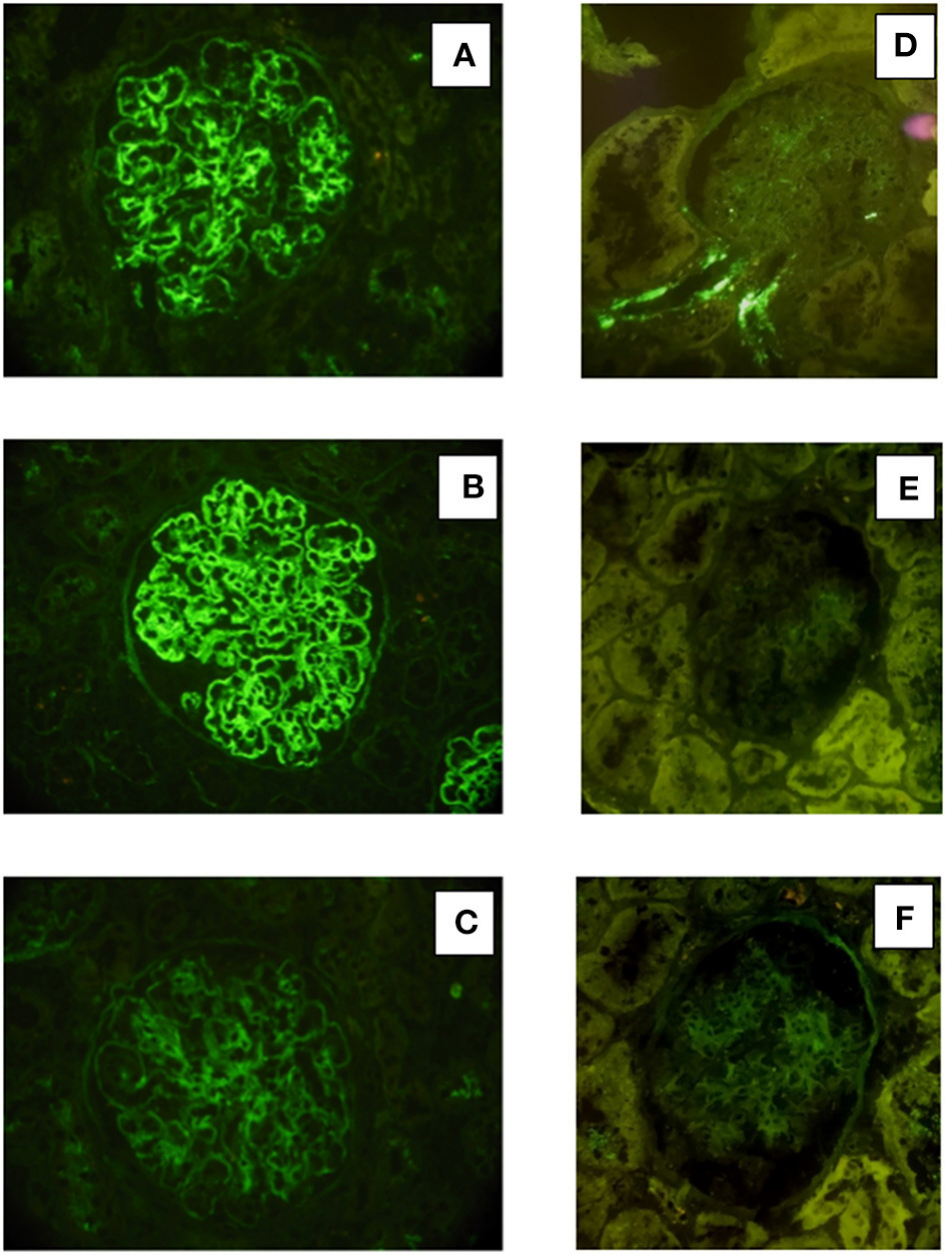
Immunofluorescence findings in patient #1: the first biopsy showed bright positivity (3+) for C3 **(A)** and k-light chain **(B)** involving the glomerular capillary walls and the mesangium, while it resulted negative for λ-light chain **(C)**. The repeat biopsy showed substantial negative staining for C3 **(D)** and k and λ -light chain **(E, F)**.

**Figure 3:**
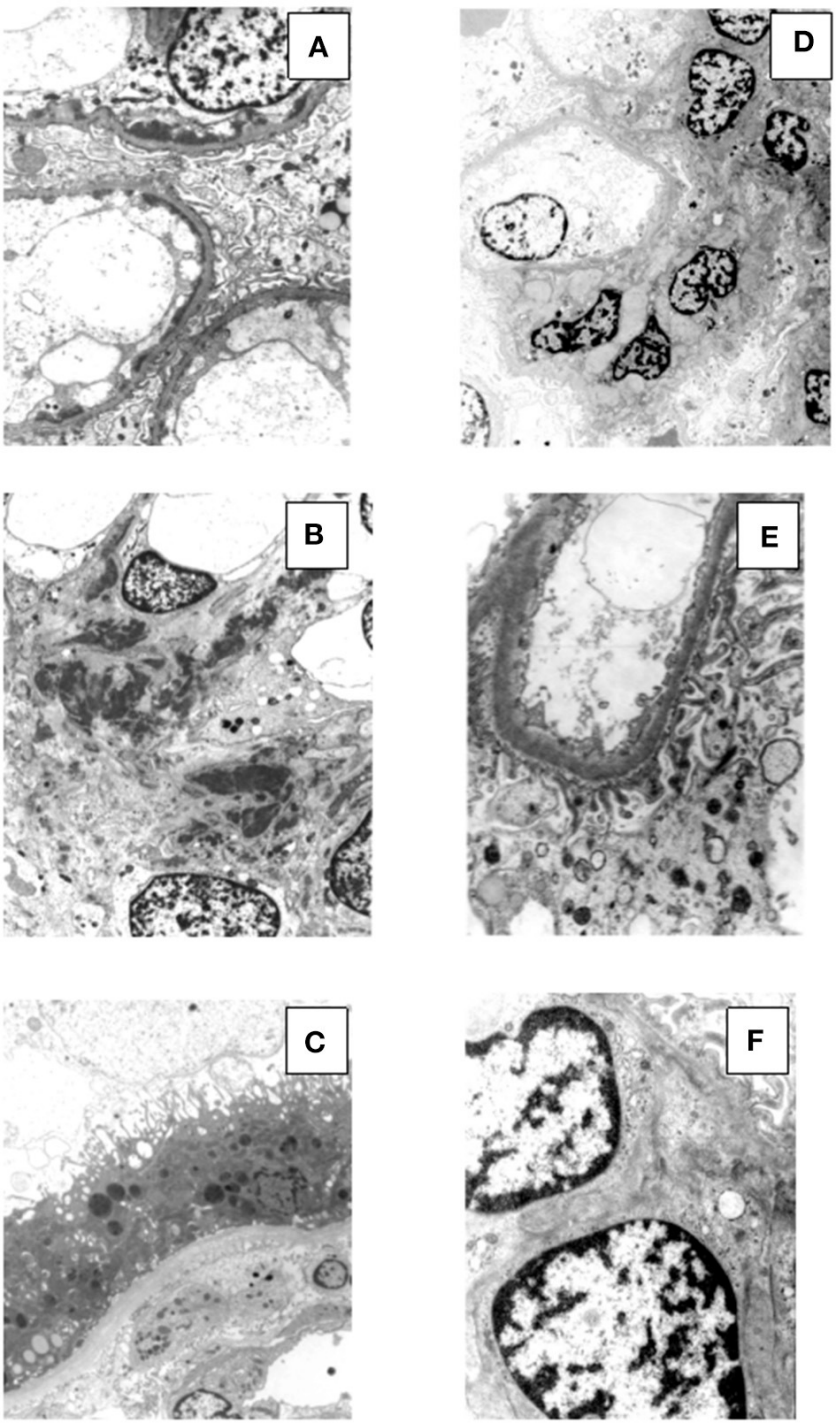
Electron microscopy findings in patient #1: the first biopsy showed subendothelial (**A**, original magnification, x3900) and mesangial (**B**, original magnification, x2950) electron dense deposits, while no deposits were seen along the tubular basement membranes (**C**, original magnification, x2950). The repeat biopsy showed complete disappearance of the subendothelial and mesangial deposits. (**D**, original magnification, x2200; **E**, original magnification, x6610; **F**, original magnification, x8900).

Renal tissue specimens were examined by light microscopy, immunofluorescence, and electron microscopy. Light microscopy showed membranoproliferative glomerulonephritis with nodular mesangial expansion, and infiltrating mononuclear and polymorphonuclear leukocytes (Figures 1A–C). Immunofluorescence showed positive staining for C3 (3+) and k-light chain (3+) involving glomerular capillary walls and the mesangium, with negative staining for λ-light chain and for heavy chain, performed both on frozen material (Figures 2A–C) and on formalin-fixed paraffin embedded tissue after protease digestion (not shown). Ultrastructural evaluation highlighted subendothelial and mesangial electron dense deposits, with no deposits along the tubular basement membranes (Figures 3A–C).

The patient was treated with 6 cycles (cycle length = 28 days) of cyclophosphamide-bortezomib-dexamethasone regimen (CBD, also referred to as CyBorD or VCD) and received: cyclophosphamide 350 mg per os on days 1, 8, 15 + bortezomib 1.3 mg/m2 subcutaneously on days 1, 8, 15, 22 + dexamethasone 20 mg per os on days 1, 8, 15. Then, he received 3 cycles (cycle length = 28 days) of bortezomib-dexamethasone regimen (VD): bortezomib 1.3 mg/m2 subcutaneously on days 1, 8, 15, 22 + dexamethasone 20 mg per os on days 1, 8, 15. She also received acyclovir and trimethoprim-sulfamethoxazole prophylaxis for 12 months. No adverse effects related to the cytotoxic therapy were observed.

Renal biopsy was repeated after 12 months which showed: a remarkable reduction of the membranoproliferative lesions with disappearance of the capillary leukocyte infiltration and persistent mesangial expansion (Figures 1D–F); substantial negative staining for C3 and k and λ-light chain (Figures 2D–F); complete disappearance of the subendothelial and mesangial deposits on ultrastructural examination (Figures 3D–F).

The laboratory investigations at 12 and 24 months showed substantial renal function improvement, and normalization of proteinuria, serum k/λ free light chain concentration, and hemoglobin levels (Table 1).

### Case 2

A 71 year old man with a history of recent onset hypertension presented to the hospital for evaluation of moderate renal failure (eGFR = 37 ml/min per 1.72 m2) and proteinuria (1.5 g/d) discovered 4 months earlier.

On presentation, he was mildly tachypneic (oxygen saturation 90% while breathing ambient air) and had moderate lower limb edema. Laboratory investigations showed (Table 1): severely reduced eGFR (10 ml/min per 1.73 m2), nephrotic proteinuria (7.0 g/d) and microscopic hematuria with red blood cell casts in the urine sediment; mild hypocalcemia; normal complement levels; negative cryoglobulins; moderately reduced total protein and immunoglobulin G levels; normocytic anemia (hemoglobin, 10.5 g/dl); a small amount (<10%) of λ monoclonal immunoglobulin on serum immunofixation (k/λ ratio = 2.1), with negative urine immunofixation.

Real-time ultrasound-guided percutaneous renal biopsy was performed, followed by bone marrow aspirate and biopsy.

**Figure 4:**
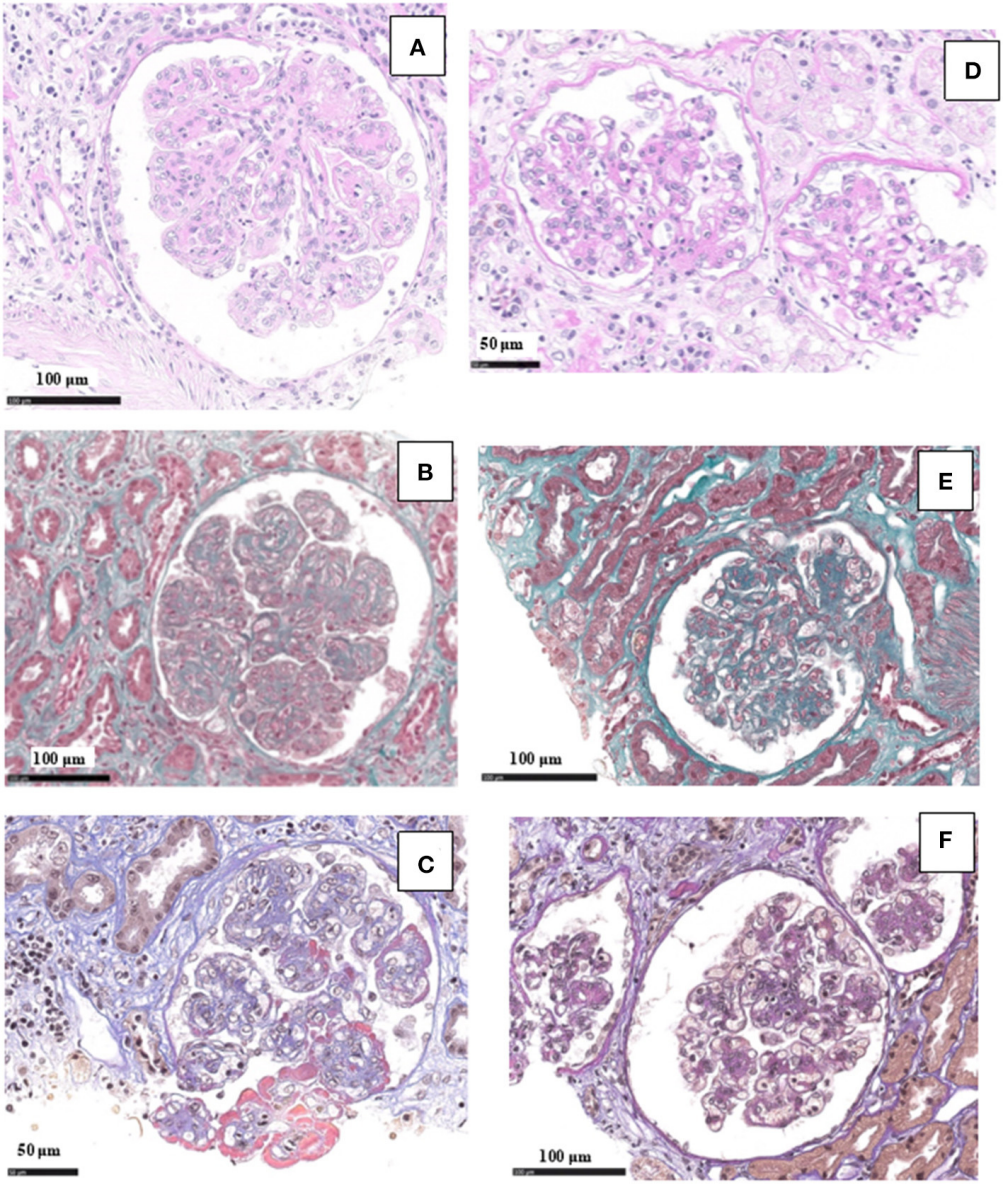
Light microscopy findings in patient #2: the first biopsy showed membranoproliferative glomerulonephritis with nodular mesangial expansion, and infiltrating mononuclear and polymorphonuclear leukocytes (**A**, PAS; **B**, Masson Trichrome; **C**, wide deposits along the glomerular basement membranes appear pink on AFOG staining). The repeat biopsy showed a remarkable reduction of the membranoproliferative lesions with disappearance of the capillary leukocyte infiltration and persistent mesangial expansion (**D**, PAS; **E**, Masson Trichrome; **F**, small mesangial deposits on AFOG staining).

**Figure 5:**
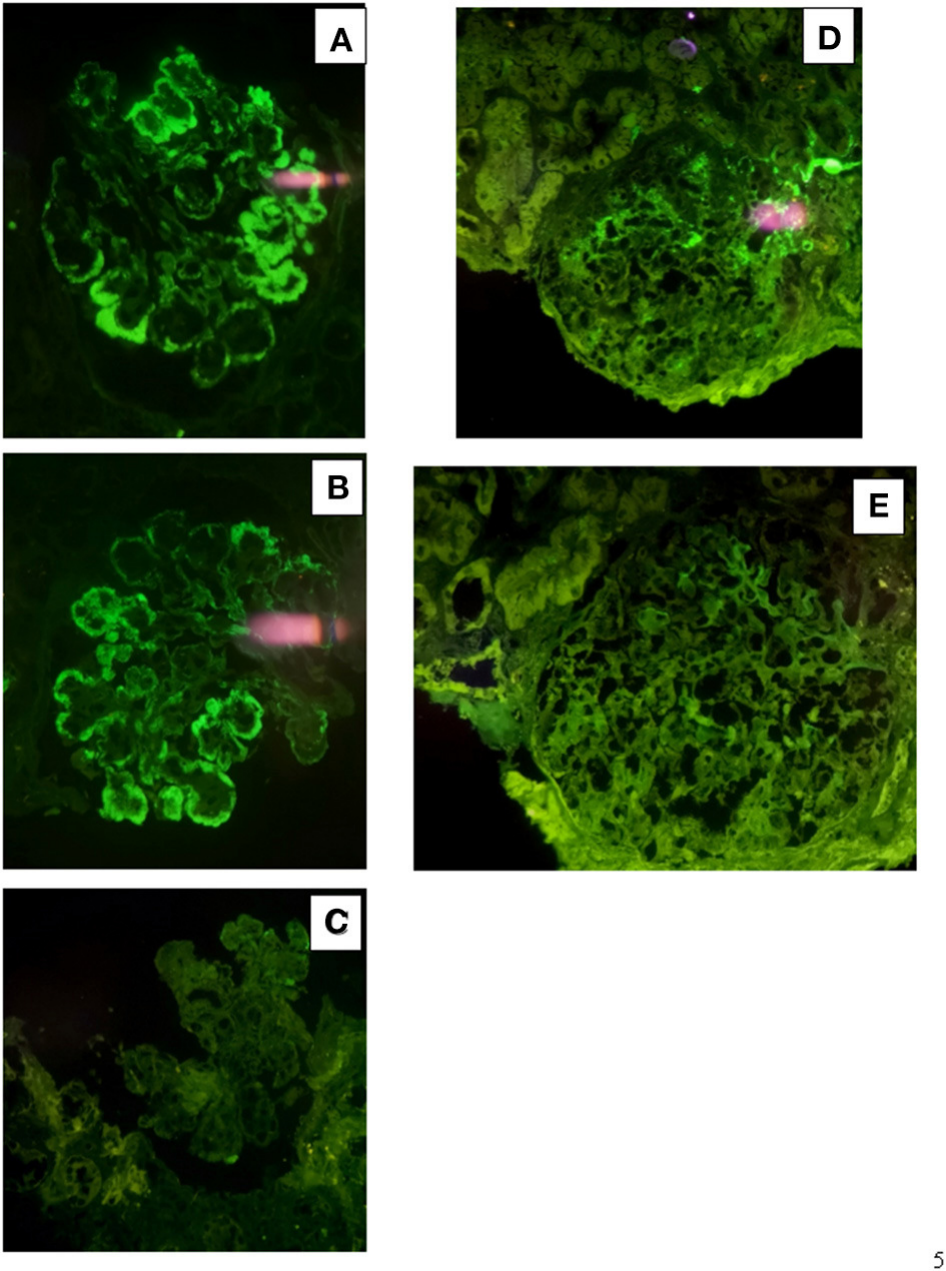
Immunofluorescence findings in patient #2: the first biopsy showed positive, coarsely granular, staining for C3 (3+), IgG (2+/3+, **A**), C1q (1+) and λ-light chain (3+, **B**) involving glomerular capillary walls and, occasionally, the mesangium, while it resulted negative for k-light chain **(C)**. The repeat biopsy showed less evident glomerular capillary wall and mesangial staining for C3 (2+, **D**) with complete disappearance of λ-light chain deposition **(E)**.

**Figure 6:**
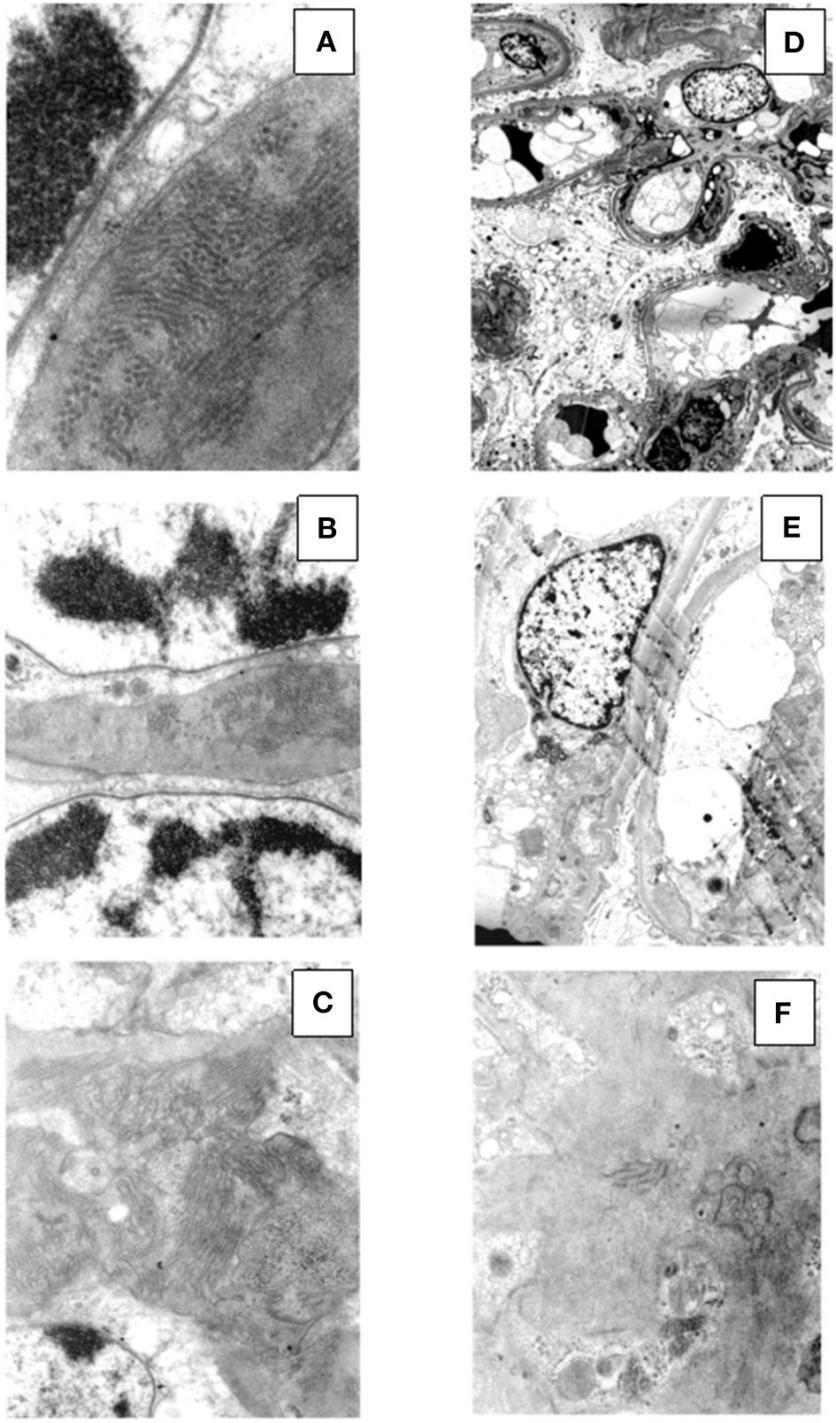
Ultrastructural findings in patient #2: the first biopsy showed large subendothelial, intramembranous, and mesangial electron dense deposits composed of microtubules (27–30 nm) with hollow centers, organized in parallel arrays (**A**, original magnification, x39000; **B**, original magnification x15500; **C**, original magnification, x15500). The repeat biopsy showed the nearly complete disappearance of the subendothelial, intramembranous and mesangial electron dense, microtubular deposits (**D**, original magnification, x1650; **E**, original magnification, x3900; **F**, original magnification, x11500: small occasional residual deposits).

Renal tissue specimens were examined by light microscopy, immunofluorescence, and electron microscopy. Light microscopy showed membranoproliferative glomerulonephritis with nodular mesangial expansion, occasional protein thrombi and numerous endocapillary infiltrating mononuclear and polymorphonuclear leukocytes (Figures 4A–C). Immunofluorescence demonstrated positive, coarsely granular, staining for C3 (3+), IgG (2+), C1q (1+) and λ-light chain (3+) involving glomerular capillary walls and, occasionally, the mesangium, with negative staining for k-light chain (Figures 5A–C). Ultrastructural evaluation showed large subendothelial, intramembranous, and mesangial electron dense deposits composed of microtubules (27–30 nm) with hollow centers, organized in parallel arrays (Figures 6A–C) consistent with immunotactoid glomerulonephritis.

Bone marrow aspirate and biopsy were examined by light microscopy, immunohistochemistry, and flow cytometry, and only showed 6% λ-restricted plasma cells.

The patient was treated with four cycles (cycle length = 28 days) of cyclophosphamide-bortezomib-dexamethasone regimen and received: cyclophosphamide 200 mg per os on days 1, 8, 15 + bortezomib 1.3 mg/m2 subcutaneously on days 1, 8, 15, 22 + dexamethasone 20 mg per os on days 1, 8, 15. Then, he received 5 cycles (cycle length = 28 days) of bortezomib-dexamethasone regimen: bortezomib 1.3 mg/m2 subcutaneously on days 1, 8, 15, 22 + dexamethasone 20 mg per os on days 1, 8, 15. He also received isoniazid prophylaxis for 6 months due to a positive QuantiFERON-TB Gold test result and acyclovir prophylaxis for 12 months. No adverse effects related to the cytotoxic therapy were observed.

Renal biopsy was repeated after 12 months and showed a significant reduction of the membranoproliferative lesions, disappearance of the capillary leukocyte infiltration and of the protein thrombi, and persistent but less evident nodular mesangial expansion (Figures 4D–F). On immunofluorescence there was less evident glomerular capillary wall and mesangial staining for C3 (2+) with complete disappearance of λ-light chain deposition (Figures 5D,E). Electron microscopy demonstrated nearly complete disappearance of the subendothelial, intramembranous and mesangial electron dense, microtubular deposits (Figures 6D–F).

The laboratory investigations at 12 and 24 months showed substantial renal function improvement, and normalization of proteinuria, serum k/λ free light chain concentration, and calcium levels (Table 1).

## Discussion

The clinical presentation of MGRS is broad and encompasses variable combinations of proteinuria, hematuria, acute or chronic renal insufficiency, hypertension, and hypocomplementemia (2, 4). Similarly, the histological patterns of kidney involvement in MGRS may be extremely complex and heterogeneous and can result from the direct deposition of nephrotoxic monoclonal immunoglobulin or its fragments as well as from indirect mechanisms. Therefore, according to the IKMG recommendations, kidney biopsy is mandatory for a correct diagnosis (3). Once an MGRS is diagnosed or suspected on the basis of renal pathology a detailed hematologic workup is essential to link the presence of the paraprotein to the associated renal disease and consequently to better define the most adequate therapy (7).

In this report, we present two cases of very aggressive clinical and histological MGRS successfully treated with a combined chemotherapy regimen. Renal biopsy carried out on the first patient showed proliferative glomerulonephritis with monoclonal k-light chain and C3 deposits. It should be emphasized that the texture of the staining was granular and not linear as observed in monoclonal immunoglobulin deposition disease (MIDD). This case represents monoclonal immunoglobulin-associated proliferative glomerulonephritis characterized by the presence of only monotypic light chain without associated heavy chain. The monoclonal immunoglobulin often includes heavy-chain IgG, less commonly IgM or rarely IgA, with k or λ-light chain restriction. Rarely only heavy or light chain may be evident (8). Very recently a series of 17 patients with proliferative glomerulonephritis with deposition of monoclonal immunoglobulin light chain only (PGNMID-light chain) has been described (9). In this series, by immunofluorescence, deposits were composed of restricted light chain (kappa in 71% of cases) and C3, as in our patient. Furthermore, patients with PGNMID-light chain showed higher frequency of a detectable pathogenic plasma cell clone and of abnormal serum free light chain compared to PGNMID with IgG deposits, supporting in these cases the necessity of bone marrow evaluation, including flow cytometry and immunohistochemical analysis (10). The second patient showed immunotactoid glomerulopathy. Both patients presented with renal failure and subnephrotic or nephrotic proteinuria, both had <10% plasma cells in their bone marrow biopsy, and neither of them had myeloma-defining events. In one patient a scanty amount of k free light chain was detected in urine immunofixation, and in the other a small amount of λ monoclonal immunoglobulin was found on the serum immunofixation. The case report regarding the first patient has been published in part in the Giornale Italiano di Nefrologia (11).

The treatment of MGRS is directed at the underlying B-cell or plasma cell clones and is based on a combination of various chemotherapy agents, aiming to preserve kidney function and prevent recurrence after kidney transplantation (7, 12–14). Autologous stem cell transplant may benefit selected patients (14). No formal guidelines exist for this disease and different regimens have been described. However, expert opinion/consensus-based treatment decisions can guide clinical practice (7, 15–18).

Based on the good results reported in different histological patterns of MGRS (7, 12–20), we decided to employ a bortezomib-based regimen, directed against the pathologic clone. Bortezomib is a proteasome inhibitor with a non-renal metabolism. Other proteasome inhibitors are currently available, but bortezomib has the most robust data in the treatment of MGRS. Therefore, both patients received a 9-month course of combined bortezomib-based chemotherapy. It is worth noting that patient 2 showed rapid improvement of renal function and proteinuria, so we decided to only administer four cycles of cyclophosphamide (instead of six) to reduce the risk of tuberculosis reactivation, due to his positive QuantiFERON-TB Gold test result.

The chemotherapy regimen resulted in substantial renal functional improvement, complete normalization of proteinuria and disappearance of serum and urine clones in both patients. We decided to repeat the renal biopsy after 12 months to evaluate the histological evolution of the kidney injury and to define the need for further treatment. The repeat biopsies actually showed a dramatic improvement of the proliferative lesions and the complete disappearance of the capillary wall and mesangial deposits, suggesting a very good histological response to the regimen we employed, therefore no further chemotherapy was prescribed.

The laboratory investigations at 24 months confirmed complete biochemical remission. No adverse effects were reported.

These two cases suggest that targeting plasma cell clones responsible for producing a paraprotein causing kidney injury may be an appropriate strategy when treating patients with MGRS, regardless of the histological pattern of renal disease. It is worth noting that while treatment with bortezomib-based regimens is commonly adopted in monoclonal gammopathy associated proliferative glomerulonephritis (7, 12–20), such regimens are only occasionally reported in MGRS with an immunotactoid pattern (21), where anti-CD20 therapy is frequently adopted as many cases are related to a B cell proliferative disorder. In our two patients the bortezomib-based regimen was probably very effective at least in part because of the presence of a mildly increased number of plasma cells in bone marrow biopsies.

A major strength of this report is that it is the first to include MGRS repeat renal biopsies at the end of therapy. Re-examination of histology may be useful in providing insight into the evolution of kidney injury, as well as in guiding the decision about further therapeutic approaches or, as in our two patients, in supporting discontinuation of therapy.

MGRS may be a progressive disease that, if untreated, can lead to end stage renal failure and recur after renal transplantation (2, 13, 15, 20). No randomized controlled trials exist to guide the optimal approach to therapy, but bortezomib-based regimens are frequently used.

In our two patients with aggressive renal disease, combination therapy of bortezomib, cyclophosphamide and dexamethasone proved to be extremely effective and safe.

## Data Availability Statement

The original contributions presented in the study are included in the article/supplementary material, further inquiries can be directed to the corresponding author/s.

## Author Contributions

GQ, MM, and DR were responsible for the research idea and study design. AB was responsible for the histological evaluation. MPa, MPi, and AV were responsible for the hematological follow-up and data analysis/interpretation. GDV and EDS were responsible for data acquisition. RF and MF were responsible for revising the article. Each author contributed important intellectual content during manuscript drafting or revision, accepts personal accountability for the author's own contributions and agrees to ensure that questions pertaining to the accuracy or integrity of any portion of the work are appropriately investigated and resolved.

## Conflict of Interest

The authors declare that the research was conducted in the absence of any commercial or financial relationships that could be construed as a potential conflict of interest.
